# Cellular Pharmacology of Palladinum(III) Hematoporphyrin IX Complexes: Solution Stability, Antineoplastic and Apoptogenic Activity, DNA Binding, and Processing of DNA-Adducts

**DOI:** 10.3390/ijms19082451

**Published:** 2018-08-19

**Authors:** Georgi Momekov, Iva Ugrinova, Evdokia Pasheva, Daniela Tsekova, Galina Gencheva

**Affiliations:** 1 Department of Pharmacology, Pharmacotherapy and Toxicology, Faculty of Pharmacy, Medical University-Sofia, 2 Dunav Str., BG1000 Sofia, Bulgaria; 2 Laboratory of Chromatin Structure and Functions, Institute of Molecular Biology, Bulgarian Academy of Sciences, G. Bonchev Str. Bl.21, BG1113 Sofia, Bulgaria; ugryiva@gmail.com (I.U.); eva@bio21.bas.bg (E.P.); 3 Department of Analytical Chemistry, Faculty of Chemistry and Pharmacy, Sofia University, 1 J. Bourchier Str., BG1164 Sofia, Bulgaria; dtsekova@chem.uni-sofia.bg

**Keywords:** palladium(III) complexes, hematoporphyrin IX, antiproliferative activity, DNA binding and repair, HMGB-1 protein, apoptosis

## Abstract

Two paramagnetic Pd^III^ complexes of hematoporphyrin IX ((7,12-bis(1-hydroxyethyl)-3,8,13,17-tetramethyl-21H-23H-porphyn-2,18-dipropionic acid), Hp), namely a dinuclear one [Pd^III^_2_(Hp_-3H_)Cl_3_(H_2_O)_5_]·2PdCl_2_, **Pd1** and a mononuclear metalloporphyrin type [Pd^III^(Hp_-2H_)Cl(H_2_O)]·H_2_O, **Pd2** have been synthesized reproducibly and isolated as neutral compounds at different reaction conditions. Their structure and solution stability have been assayed by UV/Vis and EPR spectroscopy. The compounds researched have shown in vitro cell growth inhibitory effects at micromolar concentration against a panel of human tumor cell lines. A DNA fragmentation test in the HL-60 cell line has indicated that **Pd1** causes comparable proapoptotic effects with regard to cisplatin but at substantially higher concentrations. **Pd1** and cisplatin form intra-strand guanine bis-adducts as the palladium complex is less capable of forming DNA adducts. This demonstrates its cisplatin-dissimilar pharmacological profile. The test for efficient removal of DNA-adducts by the NER synthesis after modification of pBS plasmids with either cisplatin or **Pd1** has manifested that the lesions induced by cisplatin are far better recognized and repaired compared those of **Pd1**. The study on the recognition and binding of the HMGB-1 protein to cisplatin or **Pd1** modified DNA probes have shown that HMG proteins are less involved in the palladium agent cytotoxicity.

## 1. Introduction

Cisplatin and the clinically accepted platinum drugs have a great importance for the cancer treatment. They have been applied in most anticancer chemotherapeutic regimens [1,2,3,4]. Intensive studies in this area date back to the mid-sixties of the 20th century, with Rosenberg’s remarkable discovery of the medicinal power of the inorganic coordination compound “nicknamed” cisplatin [1,5,6,7]. Since then, cisplatin has revolutionized cancer treatment converting the formerly fatal disease, largely curable [8]. Nowadays, cisplatin is still the most successful anticancer drug in the world, and is widely used in the treatment of a multitude of different cancers. Still, regardless of the achievements of cisplatin and the related platinum-based drugs—carboplatin—a second generation, and oxaliplatin—third generation, and those of regional use in specific countries, such as nedaplatin, lobaplatin, and heptaplatin, their application is limited [9]. The restrictions are due to their major drawbacks: effectiveness against a limited range of cancers and its intrinsic resistance, development of acquired resistance [10,11], severe side-effects [12,13], and low solubility. Hence, the efforts in the field of antitumor drugs design target introduction of new formulas possessing both a widened spectrum of chemotherapy and an improved clinical profile [3,4,9,14]. Intensive research on drug development processes during the past years shows that the invention of universal compounds active against many cancer types is a task difficult to accomplish. The right way is to develop drugs effective against a small group or a subgroup of cancers. Thus, the successful route leading to new efficient drugs inducing a better tumor response in individual patients is to design a pharmacological agent targeting specific abnormalities in particular cancer cells [4].

There are several approaches to develop new metal-based antitumor agents. Historically, the first one follows the correspondence between anticancer activity and the molecular structure of cisplatin [3,4,15]. Thus, a variety of cisplatin-similar platinum(II) complexes with different ligands have been synthesized and tested. The numerous results achieved have led to a set of rules for constructing a molecular structure that appeared to be required in order to manifest antitumor activity [7]. According to the rules, the neutral platinum complex with square-planar geometry, containing two cis-am(m)ine carrier ligands (capable of participating in a hydrogen bonding formation) and two cis-coordinated leaving groups (Cl^−^ ligands) possesses the necessary structural features for intravenous administration into the blood stream. It is considered that the complex should remain largely unchanged during circulation and after entering the cells a substitution of one or two more labile ligands occurs thereby activating the complex. This requirement underlines the necessity to render the complex inactive during the transport and determines that the ligand exchange rates should be compatible to the rates of cell division processes [1,16]. The platinum(II) complexes whose structure follows these rules represent the so-called classical platinum chemotherapeutics. Numerous investigations have been conducted in order to determine the mechanism by which these drugs carry out their anticancer action [17] and most results concerns cisplatin. In general, the main steps cover: cellular uptake by passive diffusion [18] or active transport via the copper transporters CTR1 and CTR2 [19,20]; aquation followed by activation; DNA platination and cellular processing of Pt-DNA lesion leading to apoptosis or to the cell survival. Nuclear DNA is considered the ultimate target of cisplatin and related platinum therapeutics and their capability to form bifunctional DNA crosslinks causing the DNA distortion. Thus, the platinum induced kink in the DNA molecules leads to a chain of events including protein recognition and eventual apoptosis. Unfortunately, some of the platinum compounds can be activated in biological milieu before reaching the tumor cell and they can also interact with nontarget biomolecules. Furthermore, it is clear that DNA is the ultimate pharmacological target of the platinum drugs, but the important issue here is to discern tumor cells from healthy cells. Now it is accepted that the classical platinum compounds can enter in each cell, however the healthy cells and some of the tumor cells can reverse the damage and remove the platinum compounds. Therefore, it is clear that the compounds belonging to this group cannot offer any advantages over cisplatin. Indeed, more than ten other cisplatin related derivatives are currently in clinical trials and the experimental data show [21,22] that they have not overcome considerably many of the disadvantages arising from this common structure. However, the achievement analysis in this field demonstrates that metal coordination compounds can play an important part in anticancer treatment regimes.

Another route to create new metal-based anticancer agents is focused on the mechanism of their antitumor action [23]. In this respect one could distinguish compounds capable of interacting in two different mechanisms: compounds that cause DNA distortion, here including cisplatin- similar and cisplatin dissimilar interactions [3,24,25,26] and compounds interacting with the key protein targets (including enzymes) that are selective for the specific malignancy and/or that regulate apoptosis, and/or that are responsible for cell invasion and metastasis [27,28]. The classical approach based on cell viability assay and characterization of the compounds that bind to DNA and adducts formed has been applied for more than forty years and now this is the first stage for evaluation. Nowadays, the drug design concepts are based on one side on the chemical nature of the compounds and on the other side—on the cancer cells biochemistry. Thus, the new strategies for design and synthesis result in the development of new classes of antitumor agents, the so called “classical nonplatinum metal compounds” and “nonclassical metal compounds” [3] and thus several compounds (e.g., NKP-1339 [29], NAMI-A [30], Satraplatin [31], etc.) are proposed that are on the “verge” of clinical application. These strategies are consistent with requirements for: a comparatively soluble prodrug’s form; existence of an inactivated form in the blood stream; selective cellular uptake; activation of the drug form in the tumor cells; appropriate tumor cell damage.

Metal coordination compounds with their inherent properties can meet these requirements to a great extent. Their specific three-dimensional structures combined with suitable electronic and ligand exchange properties are attractive for the creation of a successful formula [3,4]. The transition series metal complexes having variable oxidation states and coordination numbers, and the ability to bind, of different strength, to a wide variety of donor functional groups are some of the most tested compounds. A special attention is paid to the metals from second and third transition series. They usually exchange their ligands slowly, on the same time scale as the cell division processes. Their comparatively inert behavior keeps them as prodrugs, which have to be activated by ligand substitution reaction in the cells. In general, from a chemical point of view, the successful drug should be a well soluble compound with lipophilicity and acid-base properties determining a selective uptake. The drug should be activated inside the tumor cells and attack the target molecules by ligand substitution reactions. It is necessary to interact predominantly with target molecules and the formed adducts should be stable enough in order to provoke proper cell death. It means that a key element in the design processes is the control on the kinetics of ligand substitution reactions in vivo and the thermodynamic stability of the initial, intermediate, and final complexes produced during the antitumor action. In addition, the redox properties determined in terms of both metal and ligand are of special interest. As a part of antitumor mechanism, the change of the metal oxidation state can trigger ligand release and the participation of the ligands in in vivo redox reactions could produce reactive oxygen species. Also, the possibility to achieve light-triggered activation of an excited-state of the metal complexes in tumor cells instead of their ground-state gives additional advantages in the drugs design. All these considerations give the reason to conclude that a properly selected metal ion with regard to its nature, oxidation state, and coordination polyhedron with right ligands in the inner coordination sphere can create a drug able to overcome some of cisplatin’s disadvantages.

**Scheme 1:**
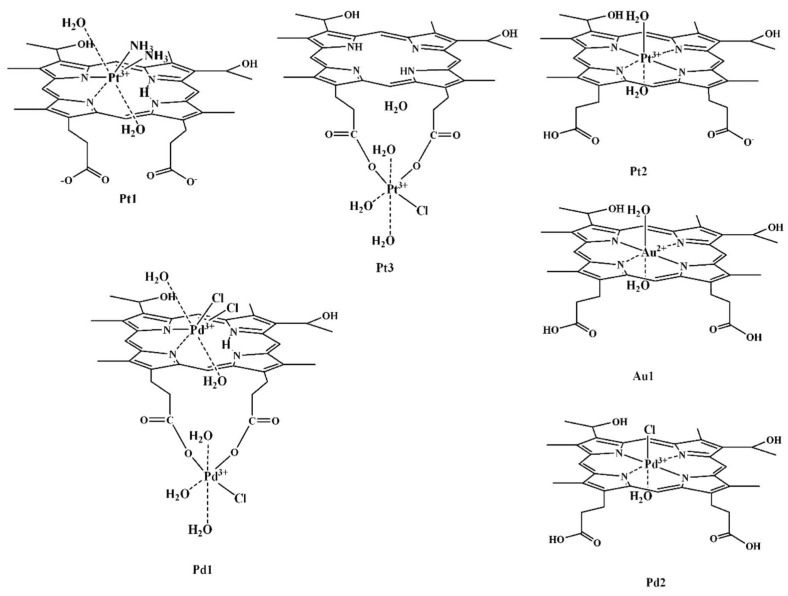
Structures of the hematoporphyrin IX complexes of platinum(III), gold(II) and palladium(III).

Recently, a series of hematoporphyrin IX (7,12-bis(1-hydroxyethyl)-3,8,13,17-tetramethyl-21H-23H-porphyn-2,18-dipropionic acid, Hp) complexes [32,33,34,35,36,37,38,39] of platinum, palladium, and gold (Scheme 1) have been tested as tumor growth inhibitors. Hematoporphyrin IX and its derivatives are well known for their widespread application in the photodynamic therapy and diagnosis [40]. It is considered that, due to their acid-base and hydrophobic properties, the porphyrins could preferably accumulate in the neoplastic tissues [41,42,43]. In fact the selective uptake of porphyrins in malignant tissue cells is due to complex mechanisms, the most important of them being the LDL-receptor mediated endocytosis of porphyrin—lipoprotein complexes formed in the systemic circulation [40].

A new strategy for cytotoxic agents design with improved properties has been proposed in our group, based on the capabilities of this ligand to stabilize unusual oxidation states of the metals. Thus, three Pt^III^ (d^7^) [32,33], two Pd^III^ (d^7^) [34], and one Au^II^ (d^9^) [35,36] complexes of Hp were obtained and characterized. The metal ions in the complexes have distorted octahedral coordination. Because of the metals’ electronic configuration, the complexes manifest paramagnetic behavior. Further they show comparatively high cytotoxicity in in vitro tests against a panel of human cell lines [32,33,34,36]. Great efforts have been made in order to understand the effect of the ligand coordination to the antitumor behavior of the complexes. The ligand Hp has a polydentate nature. Three different modes of coordination have been established [37] studying its interaction with metal ions representatives of first [38,39], second [34], and third transition series [32,33,35,36]. Coordination via the four pyrrole N-atoms in the porphyrin framework and forming of metalloporphyrin-type complexes is the most widespread mode of binding, typical of all metal ions under investigation. Coordination via two N-atoms of adjacent pyrrole rings to the metal ions in cis-position leads to the so called “sitting atop” type complexes (SAT). Coordination via the side chains deprotonated propionic COO^−^ groups, outside the porphyrin macrocycle is a less common mode of coordination. The nitrogen donor atoms of the imino (>N) and aza (=N-) groups of the pyrrole rings, as well as the outside COO^−^ groups determine the nature and the size of three different coordination modes. The metal ions choose a different mode of coordination as a function of their nature and properties.

Due to its intrinsic inertness, Pt^III^ forms stable complexes with the three different modes of Hp coordination (Scheme 1) in the proper reaction conditions [32]. All these complexes with distinct coordination patterns exert concentration dependent antiproliferative activity against a spectrum of cell lines representing some important types of neoplastic diseases in humans. They have also proven to be far less cytotoxic against the human embryonal kidney cell line HEK-293T compared to cisplatin. It has been discovered that the “sitting atop” complex **Pt1**, with PtN_4_ coordination plane formed by two adjacent porphyrin pyrrole nitrogens and two NH_3_-molecules in cis-position as well as the metalloporphyrin-type complex **Pt2** possess higher potency compared to **Pt3**. In the latter, Pt^III^ is coordinated to the deprotonated propionic carboxylic groups from the side chains of hematoporphyrin IX. The better solubility of **Pt2** compared to **Pt1** makes it preferable in research. Thus, its characteristics, such as superior proapoptotic activity that strongly correlates with its cytotoxicity and significantly higher degree of accumulation in tumor cells compared to the reference drug cisplatin, make it a reliable representative of the group of the “metal-based drugs that break the rules” [4].

Another promising representative of this group of compounds is the octahedral gold(II) (d^9^) complex of hematoporphyrin IX, **Au1** [35] that is structurally similar to **Pt2**. The comparison of its cytotoxicity to that of **Pt2** manifests that the Au^II^ complex exerts superior activity exactly against the T-cell leukaemia SKW3. These data correlate well with the estimated specific inhibiting effect of gold species upon immune cells and T-cells [36]. Nevertheless, the compound exerts well-pronounced proapoptotic properties against malignant cells and it is less cytotoxic than cisplatin for the human kidney. Furthermore, this compound demonstrated significant intracellular accumulation presumably mediated by formation of FCS-lipoprotein complexes and subsequent endocytosis.

Two relatively new members of this group compounds are the Pd^III^ (d^7^) coordination compounds of hematoporphyrin IX [34]. The dinuclear [Pd^III^_2_(Hp_-3H_)Cl_3_(H_2_O)_5_]·2PdCl_2_, **Pd1** and mononuclear [Pd^III^(Hp_-2H_)Cl(H_2_O)]·H_2_O, **Pd2** have been obtained during the interaction of the ligand with Pd^II^Cl_4_^2−^ in alkaline-aqueous medium. In the dinuclear complex, **Pd1**, one of the Pd^III^ ions is coordinated to the deprotanated COO^−^ groups from the side chains of the porphyrin ligand and the second Pd^III^ ion—to two adjacent pyrrole N-atoms on the top of the porphyrin ring. The compound is spontaneously obtained at a large metal excess from the reaction in alkaline-aqueous medium. It is accepted that because of the greater kinetic lability of Pd^III^ compared to Pt^III^ both places for coordination are occupied simultaneously and thus a dinuclear Pd^III^-Hp-Pd^III^ system is formed. The Pd^III^ ion in the mononuclear complex, **Pd2** is incorporated in the porphyrin core. The Pd^III^ centers in both complexes have a distorted octahedral coordination filled with additional donor species such as Cl^−^ and H_2_O.

As a member of the platinum group metals, palladium, with its coordination compounds, is also extensively tested for antitumor activity [44,45,46,47]. Because of lanthanoid contraction, both metals palladium and platinum in oxidation state +2 have close ionic radii. They adopt a square-planar geometry and behave like soft acids, forming strong bonds with nitrogen and sulfur-containing ligands [48]. While the equilibrium constants, characterizing the stability of their isostructural complexes differ slightly (only about ten times higher for Pt^II^ complexes), their kinetic behavior with respect of the ligand substitution is completely dissimilar as a consequence of the lower electron density of Pd^II^. The complexes of Pd^II^ simple analogues of Pt^II^-antitumor drugs undergo aquation and ligand exchange reaction 10^4^ to 10^5^ times more rapidly. The fast hydrolysis of the leaving groups leads to the formation of very reactive Pd^II^-aquated-species, unable to reach their pharmacological targets as active compounds. It is accepted that the toxic side effects are a result of inactivation of certain enzymes due to binding to the thiol groups of cysteine residues and obviously the much higher reaction rates typical of Pd^II^ compounds are favorable for general toxicity.

In order to tune the kinetic behavior and thermodynamic stability of the new compounds proposed as antitumor agents different approaches can be applied. Here, beside the construction of special coordination polyhedra, the intermediate oxidation state +3 of palladium centers has been applied as a factor of great importance. The less common oxidation state of palladium +3 provides many advantages of its complexes, such as a controlled delay of the ligand substitution reactions and reactivity, an octahedral geometry with axial ligands that could alter the redox potential and lipophilicity. The promising antiproliferative activity in micromolar concentration range that has been shown by the Pd^III^-Hp complexes [34] together with the remarkable cytotoxicity against the K-562 cells with more than 4 fold lower IC_50_ value compared to cisplatin, characteristic for the dinuclear compound **Pd1**, is the basis for further detailed biological investigation on the mechanism of action of these new proposed cisplatin-dissimilar agents.

## 2. Results

### 2.1. Synthesis and Characterization in Solution

**Figure 1:**
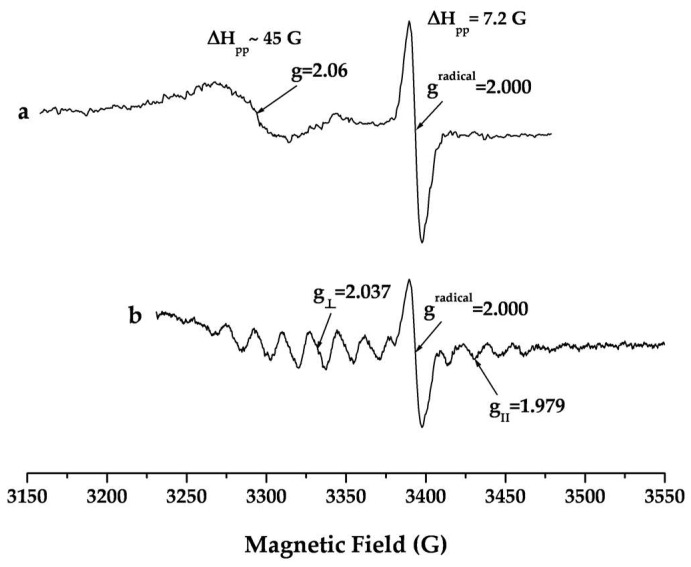
X-band EPR spectra of frozen solutions of the reaction system Pd:Hp = 1 (130 K): (**a**) a day from the beginning of the reaction; (**b**) a week later (operating frequency 9.500 GHz).

The Pd^III^-complexes that undergo extensive biological screening have been obtained from the interaction of the initial palladium(II) salt ([Pd^II^Cl_4_^2−^]) and hematoporphyrin IX. The coordination reaction proceeds in alkaline-aqueous medium on air. The ligand used for the syntheses is dissolved in 5 × 10^−2^ M KOH solution. The initial acidity of the reaction system has been adjusted within the range of pH values from 11.2 to 11.5 (by adding KOH). The reactions always start by spontaneous increase of the reaction acidity (ΔpH = 3–4) within 3 to 4 h. The creation of paramagnetic complex species during the interaction has been followed by the EPR method. The EPR spectra were registered of samples taken from the reaction systems and frozen. A very wide signal (ΔHpp ~ 250 G) with g ~ 2.12 has been observed in the EPR spectrum of the 1:1 molar ratio reaction system during the first few hours. Further, the interaction continues with appearance of two different signals of variable intensities (Figure 1a): a narrow signal (ΔHpp = 7.2 G) with g = 2.000, typical for a radical formation and a wide low-intensive signal (ΔHpp ~ 45 G) with g ~ 2.06 without a hyperfine structure. Two days later, together with a radical’s singlet, an additional third signal was observed with increasing intensity displaying a hyperfine structure. The final spectrum registered (Figure 1b) consists mainly of two signals: a singlet with parameters proving the presence of a stable radical overlaying a signal of two-component axial anisotropy and principal values of the g-tensor determined by the experiment as g_⏊_ = 2.037 and g_II_ = 1.979. The parameters measured for the superhyperfine coupling tensor are a_⏊_ = 17.8 × 10^−4^ cm^−1^ and a_II_ = 14.8 × 10^−4^ cm^−1^.

The spectral changes in the electronic-absorption spectra during the interaction in alkaline-aqueous medium refer to the bands of the free ligand spectrum as follows [49]: an intensive band belonging to a transition S_0_ ⇒ S_2_ at 376 nm (B or Soret) and four Q-bands at 505, 540, 567, and 621 nm from the transitions to nondegenerated orbitals of the first exited state S_1_, with intensities IV > III > II > I, typical of etio-type porphyrins. Two separate stages during the interaction can be distinguished. A slight hypsochromic shift of the Soret band has been observed in the beginning followed by a decrease of its intensity, widening, and a batochromic shift by ~15 nm. During this period of the reaction, the four Q-bands have been shifted batochromically. They have changed their intensities as IV > III ~ II > I and have reduced to three (~516, 558, and 620sh nm) several hours later with intensities: III ~ II > I. The UV/Vis spectra measured in the second stage of the interaction have been characterized by the Soret band at 390 nm and two Q-bands at 520 and 559 nm; the long-wave one being the more intensive. Regardless of the metal-to-ligand ratios, in the final spectrum of the interaction, the Q-bands have always reduced to two.

**Figure 2:**
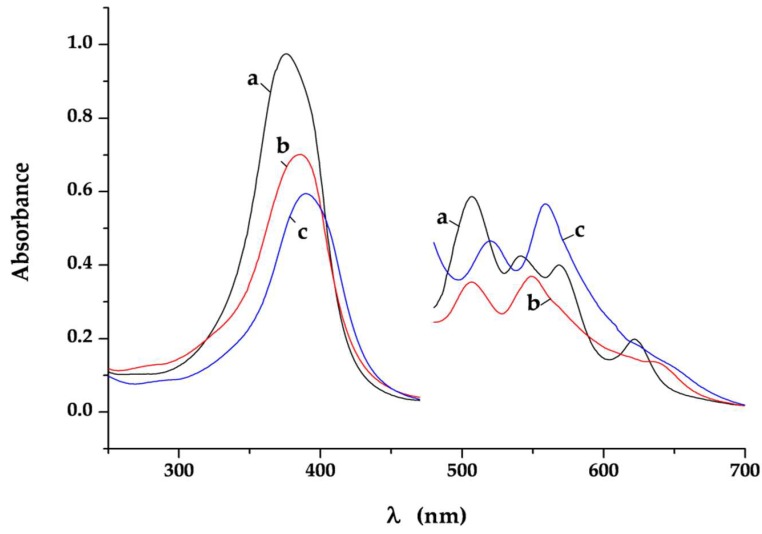
Electronic absorption spectra of: (**a**) Hp; (**b**) reaction system Pd:Hp = 4 before isolating complex **Pd1**; (**c**) reaction system 1:1 before isolating complex **Pd2**.

The reaction has been directed to obtaining the particular Pd^III^-complexes as neutral compounds at different metal-to-ligand ratios. The electronic absorption spectra of the reaction systems before the isolation of the complexes are presented on Figure 2.

A dinuclear Pd^III^ complex with a composition [Pd^III^_2_(Hp_-3H_)Cl_3_(H_2_O)_5_]·2PdCl_2_, **Pd1** is the main product of the interaction at a metal excess (Pd:Hp ≥ 4). The compound was separated spontaneously from the reaction system with coprecipitated 2 molecules of the initial PdCl_2_ at pH ~ 8. A mononuclear complex with a composition [Pd^III^(Hp_-2H_)Cl(H_2_O)]·H_2_O, **Pd2**, was the main product from the interaction at equimolar ratio of the reagents or a slight excess of one of the reagents. The compound was isolated as a neutral one by addition of hydrochloric acid (5 × 10^−2^ M HCl). The solid-state structure of the complexes and the mode of ligand coordination were studied in detail using magnetic measurements, EPR spectra, thermal and elemental analyses, and IR and UV/Vis spectroscopy [34].

**Figure 3:**
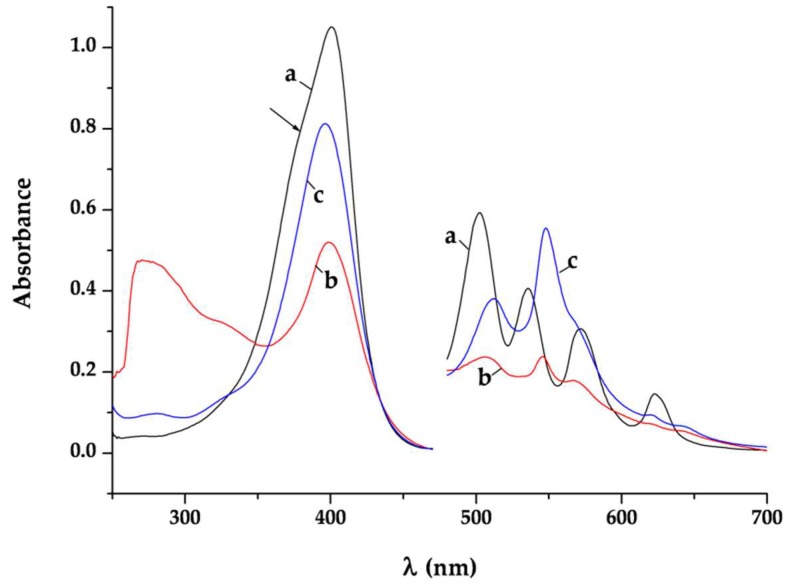
Electronic absorption spectra of Hp and its complexes dissolved in DMSO: (**a**) Hp; (**b**) complex **Pd1**; (**c**) complex **Pd2**.

The EPR and UV/Vis spectra were also used to characterize the solution-structure of the complexes and to assay their stability. Both complexes are slightly soluble in water and their solubility is pH dependent. Moderate solubility was achieved by increasing the pH value with KOH or using solvents such as DMSO (dimethyl sulfoxide) or DMF (*N*,*N*-dimethyl formamide). In the UV/Vis spectrum of hematoporphyrin IX recorded in DMSO, the Soret band (400 nm, lgε = 5.02) with a shoulder (376sh, lgε = 4.89) and the four Q-bands (502 nm, lgε = 3.77; 536 nm, lgε = 3.61; 572 nm, lgε = 3.84; 623 nm, lgε = 3.17) can be readily distinguished (Figure 3a). The UV/Vis spectra of DMSO solutions of the complexes display the characteristic three component (for Pd1: 507 nm (lgε = 3.68), 546 nm (lgε = 3.68), 567 nm (lgε = 3.63)) and two component (for **Pd2**: 512 nm (lgε = 3.58), 548 (lgε = 3.74)) pattern in the region of Q-bands (Figure 3b,c).

**Figure 4:**
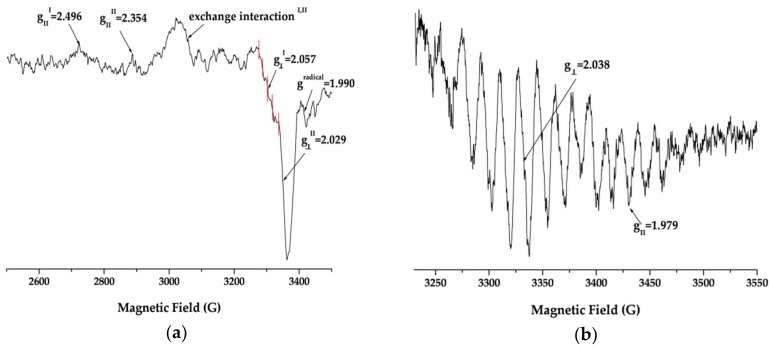
X-band EPR spectra of Hp complexes dissolved in DMSO, frozen solutions at 130 K (**a**) **Pd1**, operating frequency 9.513 GHz; (**b**) **Pd2**, operating frequency 9.502 GHz.

The EPR spectrum of the DMSO solution of the complex **Pd1** (Figure 4a) contains more than two signals. Two of them possess axial symmetry with principal values of g-tensors, respectively for the first: g_⏊_ = 2.057 and g_II_ = 2.496 with readily observed superhyperfine structure in perpendicular region (a = 15.31 × 10^−4^ cm^−1^) and for the second—g_⏊_ = 2.029 and g_II_ = 2.354. The wide signal at ~3050 G was assigned to an exchange interaction of two paramagnetic centers in the molecule. In addition, a low intensive signal with g = 1.990 and H_pp_ = 8 G was assigned to a stable radical. The EPR spectrum of the complex **Pd2** dissolved in DMSO (Figure 4b) shows only a signal with two-component axial anisotropy and principal values of the g-tensor measured from the experiment as g_⏊_ = 2.038 and g_II_ = 1.979. The superhyperfine structure measured from the experimental spectrum is well resolved both in perpendicular and parallel regions. The determined principal values of the axial superhyperfine coupling tensor are a_⏊_ = 16.65 × 10^−4^ cm^−1^ and a_II_ = 13.85 × 10^−4^ cm^−1^.

### 2.2. In Vitro Tumor Cell Growth Inhibition

The cell growth inhibitory effects of the complexes subjected to detailed biological screening were evaluated in a panel of human tumor cell lines with distinct cell type and origin in order to determine the most sensitive class of cell lines. The cytotoxicity was assessed using standard MTT-dye reduction assay [50,51]. The pilot studies covered the cell lines: SKW-3 (T-cell leukemia), LAMA-84 and K-562 cells (chronic myeloid leukemia), and 5637 (urinary bladder cancer) and the data were published in a previous research [34]. In addition, several leukemic and solid cell lines have been explored, namely: HL-60 (Acute myeloid leukemia), HD-MY-Z (Hodgkin-lymphoma), EJ (Urinary bladder carcinoma), and MCF-7 (Mammary gland carcinoma). The tested compounds inhibited the growth of tumor cells in a concentration-dependent manner. The IC_50_ values were calculated using nonlinear regression analysis and are summarized in Table 1. Normally, the tested palladium compounds exert lower activity compared to that of the referent drug cisplatin. The results showed also that as a rule the dinuclear complex **Pd1** demonstrated superior activity in contrast to the metalloporphyrin type complex **Pd2** (Table 1). Furthermore, it should be underlined, that **Pd1** exerts remarkable activity against K-562 cells, as the compound tested caused 50% cell growth inhibition at more than 4-fold lower concentration with regard to the reference agent [34]. Noteworthy, its activity against the HD-MY-Z cell line is comparable to that of cisplatin.

### 2.3. Induction of Apoptosis

**Figure 5:**
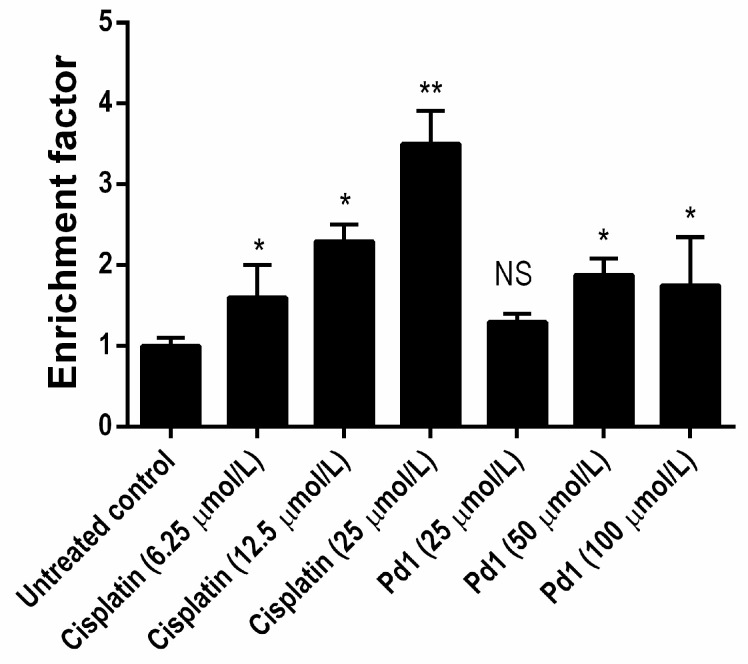
Apoptotic DNA-fragmentation in HL-60 cells after 24 h exposure to complex **Pd1** and cisplatin. The cytosolic mono- and oligo-nucleosomzal enrichment was determined using a commercially available ELISA kit. Each bar is representative for three independent experiments. Asterisks indicate statistical significance vs. the untreated control with *p* ≤ 0.05 (*) or *p* ≤ 0.01 (**) taken as significance levels; NS = non-significant (paired Student’s *t*-test).

The ability of the palladium complexes to induce programmed cell death was evaluated using the more active complex **Pd1** compared to cisplatin in HL-60 cells. The assessment has been made with a commercially available DNA-fragmentation kit allowing semi-quantitative determination of the degree of oligonucleosomal genomic DNA fragmentation. The results concerning the HL-60 cell line (Figure 5) proved that both, **Pd1** and cisplatin cause significant increase in the apoptotic histone-associated DNA fragments, but the comparison of their behavior shows that the novel dinuclear compound causes comparable proapoptotic effects at substantially higher concentrations, which is in line with the tumor cell line chemosensitivity bioassay (Figure 5).

### 2.4. DNA-Modification

**Figure 6:**
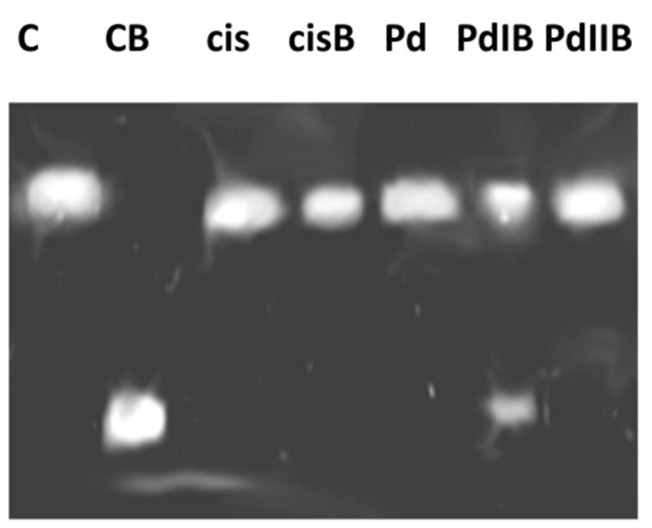
Metallation of a 40-base DNA fragment following treatment with cisplatin (molar ratio 1:50) and **Pd1** (molar ratios 1:100 and 1:200). The level of metal binding following interactions between the tested complexes and the DNA probe was analyzed after BamH1 treatment, electrophoretic analysis in 5% native polyacrilamide gel, and ethidium bromide staining. On the picture: **C**, unmodified DNA; **CB**, BamH1-digested DNA; **cis**, cisplatin-modified DNA; **cisB**, cisplatin-modified DNA (1:50) BamH1-digested; **Pd**, **Pd1**-modified DNA; **PdIB**, **Pd1**-modified DNA (1:100) BamH1-digested, **PdIIB**, **Pd1**-modified DNA (1:200) BamH1-digested.

In order to evaluate in parallel the ability of **Pd1** and cisplatin to form intra-strand guanine bis-adducts, we investigated their binding to a synthetic 40-base DNA fragment bearing a single GG sequence [52]. Optimal binding to the target DNA molecule was observed at molar ratio 1:50 for cisplatin and molar ratio 1:100 for **Pd1**, respectively, after overnight incubation (Figure 6). Cisplatin treatment led to a total inhibition of the BamH1-mediated fragmentation of the DNA-probe indicating a high metallation ability. The dinuclear palladium complex **Pd1** also inhibited the nuclease activity as is evident by the low quantity of digested fragment but failed in the totally hamper the fragmentation of the target DNA-molecule. 

### 2.5. DNA-Repair Synthesis

**Figure 7:**
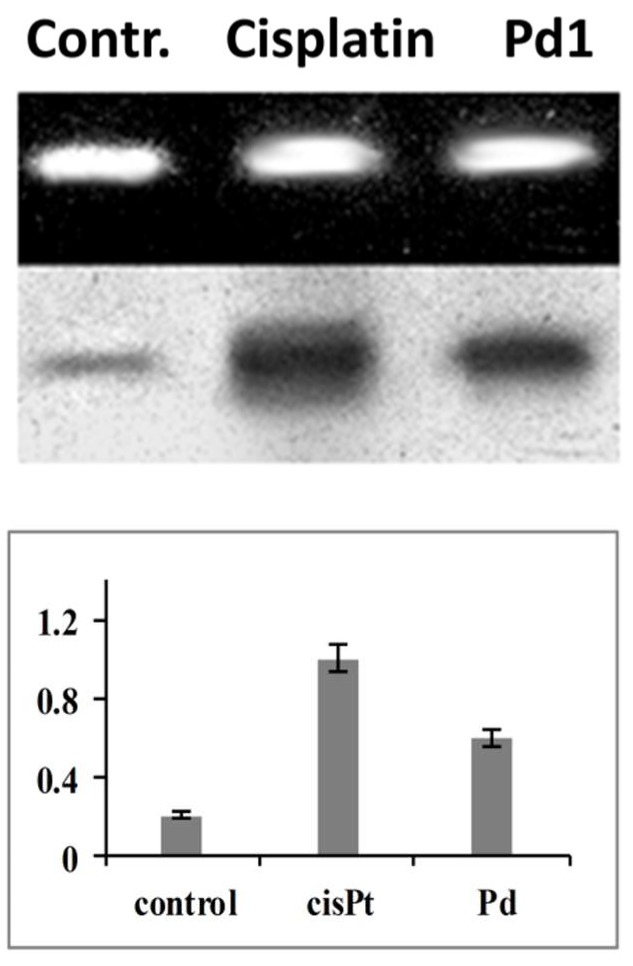
Relative efficiency of the nucleotide excision repair process in pBS plasmids modified by treatment with cisplatin or **Pd1** (plasmid:drug ratio 1:750, overnight). Repair of cisplatin-modified pBS is set as 1.

The elucidation of the cellular processing of metallodrug-induced DNA-adducts is of crucial importance for delineation of the pharmacological behavior of the antitumor agents [53,54]. The results of the DNA-repair experiments run under optimal conditions: plasmid:drug ratios 1:750, overnight are shown on Figure 7. The adducts induced by cisplatin or **Pd1** treatment were recognized and efficiently repaired by the nucleotide excision repair (NER) enzymes. It was found that the level of DNA-repair of pBS plasmid after treatment with **Pd1** was far less efficient than that encountered by the cisplatin-modified DNA probe. These findings indicate that the **Pd1** would have advantageous behavior against tumor cells, characterized by an overexpression of the NER-enzymatic machinery, mediating one of the most important mechanisms of post-target resistance to platinum drugs.

### 2.6. HMGB-1 Binding to Pd1 or Cisplatin-Modified DNA

**Figure 8:**
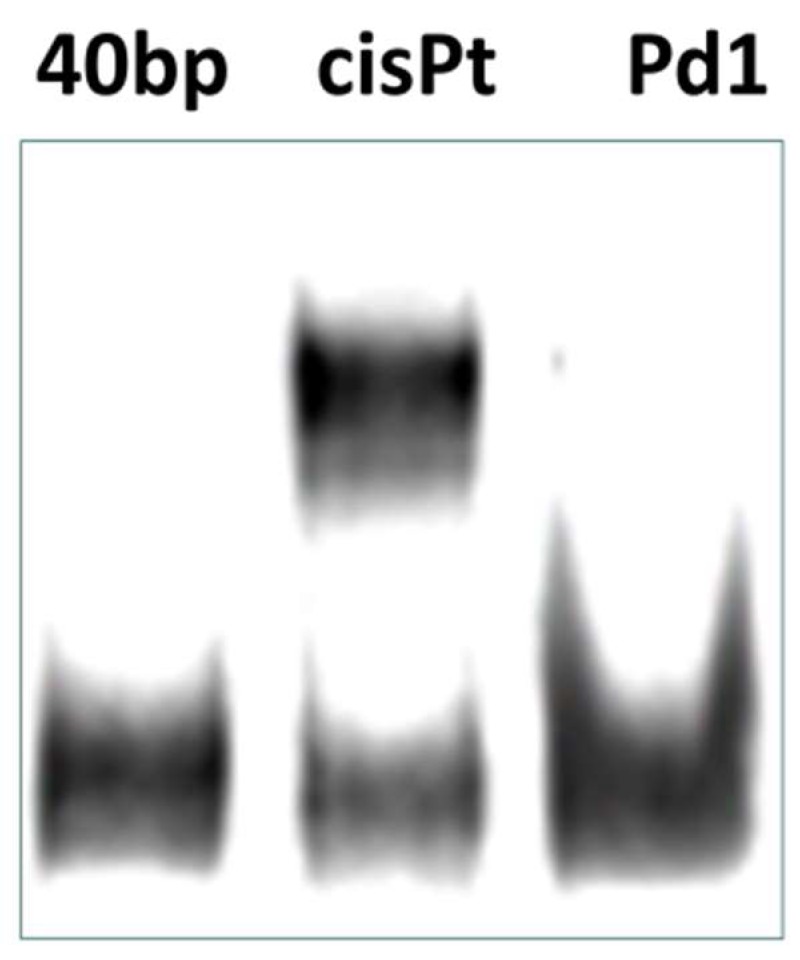
Binding of HMGB-1 protein to DNA-modified by either cisplatin or **Pd1** as determined by electrophoretic mobility shift assay (EMSA). DNA:protein molar ratio 1:9.

The ability of high mobility group box (HMGB)-1 protein to bind metallated 40-base DNA fragments was investigated in a cell-free system by electrophoretic mobility shift assay (EMSA). The modification of the DNA probe with the reference cytotoxic drug cisplatin resulted in the emergence of a more slowly migrating band indicating the specific binding of HMGB-1 to the platinated 40-base DNA fragments [55,56] (Figure 8). As seen on the electrophoregram depicted in Figure 8 of the DNA probe modification with the reference cytotoxic drug cisplatin resulted in the emergence of a more slowly migrating band indicating the specific binding of HMGB-1 to the platinated 40-base DNA fragments. In a dissimilar manner, the **Pd1**-modified DNA was not recognized as evidenced by the similar mobility of both **Pd1**-lesioned and untreated DNA probes. Thus it would be expected that the **Pd1**-modified DNA would not be shielded by the HMGB-1, indicating a further discrepancy in the molecular pharmacology of the presented class of agents vs. the classical cisplatin analogs.

## 3. Discussion

In the framework of the design of new antitumor agents, two paramagnetic Pd^III^—complexes of hematoporphyrin IX were obtained and biologically tested as representatives of the group of cisplatin-dissimilar metal-based coordination compounds. The reaction conditions for their synthesis were chosen to provide obtaining of Pd^3+^ species [57] and their stabilization in solution and solid state through formation of Hp-complexes [32,33,34,35]. The complexes were synthesized during the interaction of an initial Pd^II^ compound as a chloride complex ([PdCl_4_^2−^]) with the twofold deprotonated at peripheral propionic acid groups hematoporphyrin IX ligand ([Нр_-2Н_]^2−^) in alkaline-aqueous medium achieved by adding KOH. All measurements performed during the interaction manifested that a redoxy process takes place together with the coordination reaction. The large spontaneous increase of the acidity of the reaction system together with the appearance of the EPR signals (Figure 1) proved that the studied process is a very complicated coordination reaction with production of a mixture of paramagnetic metal complexes and stable radicals. The appearance of two-component anisotropic EPR signals with different parameters during the interaction indicates also formation of complex species with different inner coordination sphere of the paramagnetic metal centers. The mode of Hp coordination can be distinguished by using UV/Vis characterization of the reaction system. Coordination via the side deprotonated propionic COO^−^ groups could be supposed because of the hypsochromic shift of the Soret band owing to the porphyrin plane distortion. The spectral changes that follow are most probably connected to a distortion of the porphyrin ring symmetry owing to the coordination through two adjacent pyrrole N-atoms and formation of lower symmetry complex species. The reduction of the Q-bands number to two at the end of the reaction is owing to degeneration of the exited state S_1_ orbitals [58] and proves the formation of a metalloporphyrin type complex (D_4h_ symmetry).

Two of the complex species formed during the interaction PdCl_4_^2−^-Hp_-2H_ have been isolated at proper reaction conditions. A dinuclear compound **Pd1** was the main product from the interaction at a metal excess (Pd:Hp ≥ 4) and was spontaneously precipitated at pH ~ 8 and a mononuclear metalloporphyrin type complex **Pd2** was the main product of the interaction at an equimolar ratio of the reagents and was precipitated by adding hydrochloric acid (5 × 10^−2^ M HCl). The composition of the complexes was derived from the elemental analyses and the content of H_2_O and Cl^−^ in the inner or the outer coordination sphere was proven by studying their thermal behavior [34]. The molecular structure of the complexes in solid state was deduced based on detailed investigations of their magnetic properties and spectroscopic characterization, published in [34].

A crucial issue in developing a new drug formulation is to study the structure of the compound in solution and to establish the relationship with the solid state structure. The data presented here relate to the behavior of the complexes in DMSO solutions. The low temperature EPR spectra (Figure 4a,b) of the complexes dissolved in DMSO show characteristic EPR spectral patterns of paramagnetic Pd-compounds. The EPR spectra recorded several hours (5–8 h) after the dissolving correspond well to the solid state EPR spectra [34]. The two anisotropic EPR signals observed in the spectrum of **Pd1** possess axial symmetry. The principal values of the g-tensors of the two anisotropic signals g_II_ > g_⏊_ > 2.0023 are consistent with formation of an elongated octahedral coordination with (dz^2^)^1^-ground state. A five-component superhyperfine structure is observed only in the perpendicular region of the low-field signal. The principal value of the superhyperfine coupling tensor a_⊥_(N) = 15.31 × 10^−^^4^ cm^−^^1^ (I(^14^N) = 1) and the number of superhyperfine lines typical for interaction of the uncoupled electron with two ^14^N nuclei proved coordination of one Pd^3+^ via two of the pyrrole nitrogen donors of the Hp ligand. The absence of a superhyperfine structure from ^14^N nuclei on the upfield EPR signal is due to coordination out of the porphyrin ring through the propionic acid groups. The wide signal observed at ~3050 G could be assigned to exchange singlet–triplet interaction between the two unpaired electrons of the differently coordinated Pd^3+^-centers in the molecule of **Pd1**. Hence the EPR spectra proved the formation of a Pd^3+^-L-Pd^3+^ system where each of the Pd-ions possesses different coordination. The presence of a signal for a radical could be explained with significant delocalization of the unpaired electron density because of electron-acceptor properties and significant flexibility of the porphyrin moiety. The complex **Pd2** features a completely different EPR spectrum pattern in solution containing exactly one two-component anisotropic signal. The signal displays axial anisotropy with principal values of the g-tensor g_⏊_ > 2.0023 > g_II_ proving formation of a compressed octahedral structure with (dx^2^ − y^2^)^1^ ground state. Superhyperfine lines were observed both in perpendicular and parallel regions. The principal values of the axially symmetrical superhyperfine coupling tensor are typical for the interaction of an uncoupled electron with ^14^N (I = 1) nuclei. The nine superhyperfine lines are readily distinguished in the perpendicular region and prove coordination of Pd^3+^ into the four pyrrole N-donors in the porphyrin ring. Thus the spectrum confirms the existence of a stable metalloporphyrin type complex in solution.

It is well-known that changes in the conjugation and the symmetry of the Hp-ligand can affect the UV/Vis absorption spectra and can be used to obtain data about the mode of Hp coordination at complex species in solution [32,33,34,35,36,37,38,39]. While the metal binding through inner nitrogen atoms induces strong changes in the visible region of the spectra, the variations in the peripheral substituents causes minor changes owing to geometrical deformation of the porphyrin ring. The UV/Vis spectra were recorded of the studied compounds dissolved in DMSO. It was found that the position of the Soret band is sensitive to the processes such as acid-base equilibria and aggregation, and also to the modes of metal coordination. It is accepted that the Soret band appears at 376 nm if the propionic acid groups are deprotonated and shifts in accordance with the electronic density distribution caused by protonation and metal-coordination. The major component of the Soret band in the spectrum of the free ligand dissolved in DMSO (400 nm, lgε = 5.02) is batochromically shifted compared to the spectrum registered in alkaline-aqueous medium. The shoulder observed at 376 nm is due to the protolitic equilibrium Hp + 2Solv ⇔ [Hp_-2H_]^2−^ + 2[HSov]^+^, which is clearly shifted to the neutral Hp molecules in the weakly proton-acceptor solvent DMSO. In the spectra of the complexes this band is observed at 399 nm (**Pd1**) and 396 nm (**Pd2**), respectively. Thus it indicates that the propionic acid groups are engaged in the processes of coordination or protonation.

The spectrum pattern in the area of Q-bands is determined by the symmetry group of the porphyrin macrocycle. The two diagonally located hydrogen atoms at pyrrolic nitrogens in the molecule of the free ligand define a D_2h_ symmetry and hence a four number Q-bands spectrum. The loose of a proton gives a monoprotic ligand form. Many metal ions at proper pH value (6 < pH < 10) interact with the monoprotic ligand anion and bind with its two cis-disposed pyrrole N-donors on the top of the macrocycle forming sitting-atop (SAT) complex. The metal in these complexes is located out of the porphyrin plane and distorts it. The symmetry of the structure is lower (approximately between C_4v_ → C_1_) than that of both metalloporphyrins (D_4h_) where the two ammine hydrogens are substituted by the metal located coplanar on the porphyrin ring and the free Hp ligand (D_2h_). This reflects on the Q-bands number reduction and changes their intensities. Hence the observed spectrum of the complex **Pd1** in the area of Q-bands containing mainly three Q-bands (507, 546, and 567 nm) with intensity IV ~ III > II is consistent with coordination through the two cis-disposed pyrrole N-donors on the top of the porphyrin macrocycle substituting one of the pyrrolic nitrogen. Further on, the slight red shift of the Q-bands: IV and III and a blue shift of the Q band II with degeneration of the Q_x_ and Q_y_ orbitals connected with vibronic structure supports simultaneous coordination on the top of the porphyrin ring and out-side the porphyrin macrocycle through the peripheral deprotonated carboxylic groups. The typical two Q-band spectrum of the complex **Pd2** indicates unambiguously a D_4h_ symmetry achieved at coordination through the four *N*-heteronuclei in the porphyrin macrocycle. The intensive bands at 270 (lgε = 4.98) and 323 nm (lgε = 4.81) in the spectrum of **Pd1** as well as the one at 280 (lgε = 3.99) in the spectrum of **Pd2** were assigned to the ligand-to-metal charge transfer bands of the Pd-Cl bonds. This proves the presence of Cl^−^ ions in the palladium ions inner coordination sphere. 

The spectroscopic characteristics observed were unchangeable within more than five days. Hence in this period the compounds, dissolved in DMSO solution are paramagnetic palladium-hematoporphyrin IX complexes as follows: (1) for **Pd1**—a dinuclear Pd-Hp-Pd system, with two Pd^III^ ions that occupied two coordination places—at the porphyrin ring binding out-of-plan through two cis-disposed N-donors and at the two peripheral propionic acid groups; (2) for **Pd2**—normal metalloporphyrin of Pd^III^. Furthermote, this is the reason to accept that during the biological investigation the active species in solution are these palladium complexes of hematoporphyrin IX.

The cytotoxic screening of the two palladium(III) complexes was conducted on a wide spectrum of cell lines—representatives of the main human cancer types. The results of the MTT-dye reduction assay [50,51] unambiguously indicate that the two compounds exert concentration-dependent antiproliferative effects against the chosen spectrum of cell lines. Data analysis shows that the tested palladium compounds are generally less active than cisplatin, causing half-maximal inhibition of cell viability at generally higher concentrations (Table 1). It has also been proven that the dinuclear palladium complex **Pd1** demonstrates superior activity as compared to the metalloporphyrin type complex **Pd2**. The latter **Pd2** compound exerts only marginal activity and fails to cause 50% inhibition of malignant cell growth against a half of the cell lines under evaluation. Throughout the panel investigated the anticancer drug cisplatin proved to outclass the novel palladium complexes, with the only exception of K-562 leukemia whereby **Pd1** showed remarkable cytotoxicity [34].

These findings gave us a reason to conduct more detailed pharmacodynamic evaluation of **Pd1** especially regarding its ability to induce apoptosis and to modify DNA. Significant apoptotic fragmentation of genomic DNA was established after treatment of HL-60 cells with both cisplatin and **Pd1**, whereby the proapoptotic effect of **Pd1** required the cell exposure to concentrations significantly higher than its IC_50_. These data indicate that the cytotoxicity of both compounds is mediated by induction of apoptosis, although its threshold level was significantly higher for the palladium compound. 

Further experiments were carried out in order to characterize the adduct-forming ability of **Pd1** and the cellular processing of the DNA-lesions. The N7 position of guanine is considered as the ultimate pharmacological target of platinum drugs, leading to formation of intrastrand adducts whose recognition and processing leads to activation of the cell death signaling pathways [59,60]. On this ground we studied the metallation of a single strand 40-base DNA fragment in a cell free system. The palladium compound **Pd1** was less capable of forming DNA adducts under the experimental conditions, thus further demonstrating its cisplatin-dissimilar pharmacological properties. These findings indicate that although the DNA-modification plays an important role for the mode of antiproliferative action of **Pd1**; its capacity to modify DNA is lower as compared to that of cisplatin.

The structural perturbations induced by the DNA-metallation are recognized by diverse proteins including the DNA-repair enzymatic machinery [59,61,62]. Removal of platinum-DNA adducts by the nucleotide excision repair (NER) is one of the crucial mechanisms of cellular resistance to cisplatin. This prompted us to evaluate the efficiency of NER repair synthesis after modification of pBS plasmids with either cisplatin or **Pd1**. The lesions induced by cisplatin were far better recognized and repaired as compared to those of **Pd1**, implying that, by virtue of the significant structural differences between the tested complexes, they induce highly dissimilar alterations of DNA conformation. The lower level of NER-mediated removal and repair of **Pd1** modified DNA are an advantageous future of the novel compound as this would condition retained activity against malignant cells overexpressing the NER-enzymatic system.

Apart from the DNA-repair enzymes other proteins are also capable of recognizing and binding cisplatin-modified DNA [59,61,63]. Among these special attention has been paid to the high mobility group domain (HMG) proteins [55,59,61,64]. They are considered to play crucial role for the cytotoxicity of platinum drugs, whereby the proposed mechanisms include: (i) shielding of platinated DNA and steric hindrance against NER-mediated repair of metal-adducts; (ii) “hijacking” i.e., binding of transcription factors or other regulatory proteins to HMG-associated platinum adducts, thus deviating them from their normal targets and compromising their role in signal transduction [59,61,63]. On this ground we evaluated the recognition and binding of HMGB-1 protein to cisplatin or **Pd1** modified DNA probes. As made evident by the results obtained **Pd1**-induced modification conditions lower level of HMGB-1 binding, as compared to cisplatin-lesioned DNA. This implies that the high mobility group proteins are most probably less involved in the cytotoxicity of the palladium agent.

## 4. Materials and Methods

### 4.1. Chemicals and Tested Compounds

All chemicals used were of analytical grade and were obtained by commercial sources and used without further purification. Agarose, ethanol, DMSO, formic acid, 2-propanol, methanol, EDTA, ethidium bromide, sodium chloride, Tris hydrochloride, Triton^®^ X-100, l-glutamine were purchased from AppliChem GmbH, Darmstadt, Germany. The tetrazolium salt 3-(4,5-dimethylthiazol-2-yl)-2,5-diphenyltetrazolium bromide (MTT) and TE buffer werе supplied by Merck, Darmstadt, Germany. Fetal calf serum (FCS), powdered RPMI 1640 medium, the amino acid, sodium pyruvate, human insulin, and polyacrylamide gel were purchased from Sigma-Aldrich GmbH, Steinheim, Germany. The referent cytotoxic drug cis-DDP was used as a commercially available sterile dosage form for clinical application (Platidiam^®^, Lachema, Czech Republic). The two complexes tested, namely: *cis*-[Pd^III^_2_(Hp_-3H_)Cl_3_(H_2_O)_5_]∙2PdCl_2_ (**Pd1**) and, [Pd^III^(Hp_-2__H_)Cl(H_2_O)]∙H_2_O (**Pd2**) were synthesized, characterized and purified as previously described [34]. UV-Visible (UV-Vis) (solv. DMSO) λ_max_/nm, (logε): complex **Pd1**: 270 (4.98), 323 (4.81), 399 (5.02), 507 (3.68), 546 (3.68), 567 (3.63); complex **Pd2**: 280 (3.99), 396 (4.91), 512 (3.58), 548 (3.74).

### 4.2. Solution Stability Assays

The absorption electronic spectra were recorded on a “Carry” 100 UV-Vis spectrometer. The EPR spectra were obtained on an X-band “Bruker B-EPR 420” spectrometer at 130 K. The UV-Vis and EPR spectra were measured after dissolving the Pd-complexes samples in DMSO within the concentration intervals of (5 × 10^−^^6^) ÷ (1 × 10^−^^4^) mol/L for UV-Vis spectra and 5 × 10^−^^4^–5 × 10^−3^ mol/L for EPR spectra, respectively.

### 4.3. Cell Lines and Culture Conditions

In this study the following cell lines were used: SKW3 (T-cell leukemia), K-562 and LAMA-84 (chronic myeloid leukemia), HL-60 (acute myelocyte leukemia), HD-MY-Z (Hodgkin lymphoma), 5637, EJ (urinary bladder carcinomas), and MCF-7 (mammary gland adenocarcinoma). The cells were maintained in controlled environment—cell culture flasks at 37 °C in an incubator ‘BB 16-Function Line’ Heraeus (Kendro, Hanau, Germany) with humidified atmosphere and 5% CO_2_. They were kept in log phase by supplementing with fresh medium, two or three times a week. SKW3, K-562, LAMA-84, HL-60, HD-MY-Z, 5637, and EJ cells were grown in RPMI-1640 medium supplemented with 10% fetal bovine serum (FBS) and 2 mM l-glutamine. MCF-7 cells were grown as monolayer adherent cultures in 90% RPMI-1640 supplemented with 10% FBS, non-essential amino acids, 1 mM sodium pyruvate, and 10 mg/mL human insulin.

### 4.4. MTT-Dye Reduction Assay

The tumor cell growth inhibitory effects were assessed using the standard 3-[4-dimethylthiazol-2-yl]-2,5-diphenyl-2H-tetrazolium bromide (MTT)-dye reduction assay as described by Mosmann [50] with minor modifications [51]. Exponentially growing cells were seeded in 96-well flat-bottomed microplates (100 μL/well) at a density of 1 × 10^5^ cells per mL and after 24 h incubation at 37 °C they were exposed to various concentrations of the tested compounds for 72 h. At least 8 wells were used for each concentration. After incubation with the tested compounds 10 μL MTT solution (10 mg/mL in PBS) aliquots per well were added. The microplates were further incubated for 4 h at 37 °C and the MTT-formazan crystals formed were dissolved by adding 100 μL/well 5% formic acid (in 2-propanol). The absorption was measured using a microprocessor controlled microplate reader (Labexim LMR1) at 580 nm. The cell survival data were normalized to percentage of the untreated control and were fitted to sigmoidal dose/response curves. The corresponding IC_50_ values were calculated using non-linear regression analysis.

### 4.5. Apoptosis Assay

The typical apoptosis oligonucleosomal DNA fragmentation was examined using a commercially available “Cell-death detection” ELISA kit (Roche Applied Science, Mannheim, Germany). Cytosolic fractions of 1 × 10^4^ cells per group (treated or untreated) served as antigen source in a sandwich ELISA, utilizing primary anti-histone antibody-coated microplate and a secondary peroxidase-conjugated anti-DNA antibody. The photometric immunoassay for histone-associated DNA fragments was executed according to the manufacturer’s instructions. The results are expressed as the oligonucleosome enrichment factor (representing a ratio between the absorption in the treated vs. the untreated control samples).

### 4.6. DNA-Binding

The DNA binding of the dinuclear palladium agent and cisplatin was assessed as previously described [52]. A 40 n.b. fragment (5′ CGCTATCGCTACCTATTGGATCCTTATGCGTTAGTGTA TG 3′), whereby the GG-motif is the recognition sequence of the restriction nuclease BamH1 was used as a target DNA molecule. The level of DNA modification following interactions between the tested complex and the 40 n.b. fragment was analyzed after BamH1 treatment, electrophoretic analysis in 5% native polyacrylamide gel, and ethidium bromide staining, as previously described [52].

### 4.7. In Vitro DNA Repair

Synthetic pBS plasmids (2.69 kb) served as a DNA probe in this study. They were propagated in *Escherichia coli* and extensively purified as closed circular DNA by means of a FlexiPrep Kit (Pharmacia Biotech, Uppsala, Sweden). Modification of plasmid DNA (200 μg/mL) with the tested palladium complex or cisplatin was carried out in TE buffer (10 mM Tris, 1 mM EDTA), pH = 7.4, in the dark at 37 °C for 16 h. Following ethanol precipitation, DNA was washed twice in 70% ethanol and redissolved in TE buffer, and the superhelical form of the plasmid DNA was purified by ethidium bromide/cesium chloride gradient centrifugation. Cell-free extract (CFE) from exponentially growing Guerin ascites tumor cells was prepared using a previously described protocol [53], adapted for in vitro DNA repair studies [54] and stored at −80 °C until use. Repair of DNA lesions induced by the metal complexes was assayed as described elsewhere [52]. Briefly, the standard 50 μL reaction mixture contained 400 ng of metal complex-treated repair substrate pBS, 400 ng, 45 mM HEPES-KOH, pH = 7.8, 70 mM KCl, 5 mM MgCl_2_, 1 mM dithiothreitol, 0.4 mM EDTA, 2 mM ATP, 20 μM each of dTTP, dGTP, dATP, 2μCi [^32^P]dCTP (Amersham, 3000Ci/mmol, Amersham Bioscinces, Freiburg, Germany), 40 mM phosphocreatine, 2.5 μg of creatine phosphokinase, 3% glycerol, 20 μg of bovine serum albumin, and 80–120 μg of cell-free extract at 30 °C for 1 h. Reactions were stopped by adding EDTA to 20 mM, and mixtures were incubated for 20 min with RNase A (80 μg/mL) followed by another 20 min with proteinase K (200 μg/mL) in the presence of 0.5% SDS. Plasmid DNA was purified with phenol/chloroform (1:1) and precipitated by 2 vol of ethanol in the presence of glycogen (Stratagene, La Jolla, CA, USA, 1 mg/mL) at −70 °C. Thereafter the plasmids were linearized by digestion with EcoRI and resolved by electrophoresis in 0.8% agarose gel containing ethidium bromide (0.5 μg/mL). After electrophoresis, the gel was photographed under UV illumination, dried under vacuum, and exposed to Kodak XAR-5 film for 12 h at −70 °C. The autoradiograph was scanned with Gel-Pro Analyser (Media Cybernetics, Bethesda, MD, USA).

### 4.8. Electrophoretic Mobility Shift Assay (EMSA)

DNA binding assay of HMGB-1 and its truncated form with ^32^P-labeled cisplatinated DNA was performed as described previously [55]. In brief, nonlabeled sonicated salmon sperm DNA was added as a competitor in all experiments except those designed to determine the dissociation constants. On completion of electrophoresis the gel was dried and exposed to Amersham hyperfilm. Quantification of band densities was performed by scanning the autoradiographs with Gel-Pro Analyzer. In some assays, the reaction mixture prepared for EMSA was supplemented with CFE preincubated with nonplatinated DNA (500-fold molar excess over the platinated probe) and loaded on the gel. Isolation and purification of recombinant HMGB-1 were carried out as described elsewhere [56].

## 5. Conclusions

Two newer members of the group of paramagnetic transition metal complexes of hematoporhyrin IX [32,33,34,35,36,37,38,39] have been proposed for extended biological screening: a dinuclear [Pd^III^_2_(Hp_-3H_)Cl_3_(H_2_O)_5_]·2PdCl_2_ and a mononuclear [Pd^III^(Hp_-2H_)Cl(H_2_O)]·H_2_O. The complexes were obtained reproducibly in alkaline-aqueous medium and were isolated as neutral compounds changing the acidity of the reaction system and the M:L molar ratios. Their structure and stability were studied in DMSO solutions in details. It was found that the active species in solution are a dinuclear complex (**Pd1**) and a mononuclear (**Pd2**) complex. In the dinuclear complex **Pd1**, one Pd^III^ ion is coordinated to the deprotonated COO^−^ groups from the side chains of the porphyrin ligand and the second Pd^III^ ion—to two adjacent pyrrole N-atoms on the top of the porphyrin ring and thus a dinuclear Pd^III^-Hp-Pd^III^ system is created. Pd^III^ in the mononuclear complex, **Pd2**, is located in the plane of the porphyrin ring and thus a metalloporphyrin type complex is formed. Pd^III^ centers in both complexes have a distorted octahedral coordination filled with additional donor species such as Cl^−^ and solvent molecules.

The compounds tested manifested cell growth inhibitory effects at micromolar concentration against tumor cell lines with distinct cell type and origin. The calculated IC_50_ values proved that in general, palladium complexes exhibit lower activity compared to that of the referent drug cisplatin and as a rule the metalloporphyrin type complex **Pd2** is less active than the dinuclear compound **Pd1**. Contrarily to the general trend the **Pd1** complex exerts remarkable activity against K-562 cells, with 50% cell growth inhibition at more than 4-fold lower concentration compared to cisplatin. It also displays relatively close activity against the HD-MY-Z cell line with regard to the referent drug. The palladium complexes’ ability to induce programmed cell death was evaluated in a comparative experiment of **Pd1** and cisplatin in HL-60 cells. The two compounds cause significant increase in the apoptotic histone-associated DNA fragments. However, the novel dinuclear compound causes comparable proapoptotic effects at substantially higher concentrations and that corresponds to the tumor cell line chemosensitivity bioassay. The parallel evaluation of **Pd1** and cisplatin’s ability to form intra-strand guanine bis-adducts shows that **Pd1** also inhibits the nuclease activity, but failed to totally hamper the fragmentation of the target DNA-molecule. Hence, although the DNA-modification plays an important role for the mode of antiproliferative action of **Pd1**, its capacity to modify DNA is lower compared to that of cisplatin. The elucidation of DNA-adducts cellular processing by the NER enzymes demonstrated that the lesions induced by cisplatin were far better recognized and repaired as compared to those of **Pd1**. The lower level of NER-mediated removal and repair of **Pd1** modified DNA are an advantageous characteristic of the novel compound and that means that **Pd1** would retain the activity against malignant cells, overexpressing the NER-enzymatic system. The ability of HMGB-1 protein to bind metallated 40-base DNA fragment with the reference cytotoxic drug cisplatin resulted in a specific binding. In a dissimilar manner, the **Pd1**-modified DNA was not recognized and it could be expected that the **Pd1**-modified DNA would not be shielded by the HMGB-1. 

The data analysis of the in-depth biological study unambiguously highlights the differences in molecular pharmacology of the presented “applicants” for antitumor agents in respect to cisplatin. Moreover, the advantages of the new compounds provide grounds for joining them to the “nonclassical metal compounds” group. Their unique structure, based on the octahedral coordination of palladium(III) stabilized with a ligand with favorable properties, is a prerequisite for constructing a new formula with a potential of controlling its kinetic behavior as well as the strength of the M-L bonds of the adducts formed in the biological milieu.

## Figures and Tables

**Table 1:** Comparative antiproliferative activity of the investigated palladium complexes (**Pd1** and **Pd2**) and cisplatin in a panel of tumor cell lines after 72 h continuous exposure (MTT-dye reduction assay).

Cell Line	Cell Type	IC_50_ (μM) ^1^		
		Pd1	Pd2	Cisplatin
SKW-3 ^2^	T-cell leukemia	34.4 ± 2.2	77.9 ± 5.1	10.1 ± 1.4
K-562 ^2^	Chronic myeloid leukemia	2.1 ± 0.9	31.9 ± 4.3	9.4 ± 2.1
LAMA-84 ^2^	Chronic myeloid leukemia	40.0 ± 3.4	94.0 ± 6.2	18.3 ± 2.6
HL-60	Acute myeloid leukemia	75.2 ± 2.9	>200	8.2 ± 1.9
HD-MY-Z	Hodgkin-lymphoma	14.1 ± 4.5	>200	10.2 ± 3.4
5637 ^2^	Urinary bladder carcinoma	96.0 ± 1.1	177.2 ± 10.2	4.4 ± 1.7
EJ	Urinary bladder carcinoma	117.2 ± 6.9	>200	9.3 ± 1.8
MCF-7	Mammary gland carcinoma	79.4 ± 4.2	>200	6.5 ± 1.1

^1^ Arithmetic mean ± standard deviation of at least eight independent experiments. ^2^ Data have been published in [34].

## Abbreviations

DMSO	Dimethyl sulfoxide
DMF	*N*,*N*-dimethyl formamide
SAT	Sitting atop
DNA	Deoxyribonucleic acid
EMSA	Electrophoretic mobility shift assay
NER	Nucleotide excision repair
HMGB1	High mobility group protein B1
FCS	Fetal calf serum
MTT	Tetrazolium salt 3-(4,5-dimethylthiazol-2-yl)-2,5-diphenyltetrazolium bromide
